# The Impact of Chronic Particulate Matter Exposure on Quality‐of‐Life Outcomes After Endoscopic Sinus Surgery

**DOI:** 10.1002/alr.70051

**Published:** 2025-10-21

**Authors:** Chandler Rygalski, Nanda Nayak, Christina Dorismond, Rory Lubner, Daniel Lofgren, Ping Li, Katherine N. Cahill, Mason Krysinski, Rakesh Chandra, Justin Turner, Naweed Chowdhury

**Affiliations:** ^1^ Department of Otolaryngology—Head and Neck Surgery Vanderbilt University Medical Center Nashville Tennessee USA; ^2^ Department of Allergy, Pulmonary and Critical Care Medicine Vanderbilt University Medical Center Nashville Tennessee USA; ^3^ Department of Otolaryngology—Head and Neck Surgery University of Alabama at Birmingham Birmingham Alabama USA

**Keywords:** air pollutants, chronic rhinosinusitis, nasal polyps, quality of life

## 1 Introduction

Chronic rhinosinusitis (CRS) is among the most common chronic upper airway diseases in the United States, with evidence suggesting chronic air pollutant exposure, such as fine particulate matter 2.5 (PM_2.5_), has implications in both its development and severity [1, 2, 3, 4]. In the current study, we hypothesized that chronic PM_2.5_ exposure could adversely impact quality‐of‐life (QoL) outcomes after endoscopic sinus surgery (ESS) in CRS patients and sought to quantify this effect.

**Key Points**

- Higher 12‐month preoperative PM_2.5_ exposure may result in decreased QoL improvement for CRSwNP.
- Decreases in QoL in CRSwNP appear driven by decreases in the psychological and sleep subdomains.
- No associations between PM_2.5_ exposure and postoperative QoL outcomes in CRSsNP were identified.

## 2 Methods

Patients undergoing ESS were enrolled in a single‐center prospective observational cohort study of CRS and followed for at least 6 months. Home addresses were converted into latitude and longitude coordinates for exposure estimation using the *geocoder* tool in DeGAUSS [5]. A validated open‐source high‐resolution spatiotemporal PM_2.5_ exposure model was used to estimate daily exposure and averaged to obtain an exposure summary measure in the year prior to surgery [6]. The 22‐item Sino‐Nasal Outcome Test (SNOT‐22) was completed at baseline and subsequent follow‐up appointments at 6, 12, 18, and 24 months to allow longitudinal evaluation of QoL outcomes. Survey results were also divided into the five underlying subdomains to allow analysis by symptom group (rhinologic, extra‐nasal rhinologic, ear/facial symptoms, psychological, and sleep) [7]. Minimal clinically important differences (MCID) for each subdomain (3.8, 2.4, 3.2, 3.9, and 2.9 points, respectively) were used to evaluate for clinically meaningful changes in postoperative scores [8]. Multivariate linear regression models were used for the overall CRS cohort and the subpopulations of CRS with and without nasal polyps (CRSwNP, CRSsNP, respectively) to estimate the association between average preoperative 12‐month PM_2.5_ exposures and postoperative QoL outcomes after adjusting for sociodemographic variables known to be associated with PM_2.5_ exposure, including RUCA scores and income. All analyses were conducted in R version 4.2.1 (R Core Team, Vienna, Austria) on the Vanderbilt Advanced Computing Cluster for Research and Education (ACCRE). A prespecified alpha threshold of 0.05 was used to determine statistical significance.

## 3 Results

A total of 226 CRS patients met inclusion criteria and had sufficient follow‐up of at least 6 months, with 113 patients in both subpopulations of CRSwNP and CRSsNP (Table S1). Mean 12‐month PM_2.5_ levels for the group were estimated at 8.2 µg/m^3^, with a range of 6.5–13 µg/m^3^. The mean (SD) baseline total SNOT‐22 score for the overall CRS cohort was 43.7 (19.9), indicative of a moderately severe level of QoL impairment. The mean overall postoperative change in SNOT‐22 scores in our patient population was −21.4 ± 21.5 points, demonstrating a notable QoL improvement after ESS. The most common follow‐up length was 6 months (31%), with decreasing frequency over subsequent time intervals with 19% of the overall population having a follow‐up length of 24 months.

Results from multivariate regression modeling of SNOT‐22 total and subdomain change scores after ESS in relation to mean 12‐month PM_2.5_ levels are shown in Tables 1 and 2 for CRSwNP and CRSsNP, respectively. In CRSwNP, a significant association between PM_2.5_ exposure and the magnitude of postoperative improvement was identified (*β* = 8.25, *p* = 0.036), with higher exposure associated with less improvement in total SNOT‐22 scores. By subgroup, there were significant similar associations in the overall change in the psychological domain score (*β* = 3.68, *p* = 0.013) and overall sleep domain score (*β* = 2.57, *p* = 0.037). Notably, no statistically significant associations were observed between postoperative SNOT‐22 total or subdomain scores and 12‐month PM_2.5_ exposures in CRSsNP subgroup.

**TABLE 1:** Multivariate linear regression models of SNOT‐22 outcomes in chronic rhinosinusitis with nasal polyps.

Characteristic	*β*	SE	*t*‐test statistic	*p* value
Change in total SNOT‐22 score				
Intercept	−96.71	32.39	−2.99	**0.004**
Mean 12‐month PM_2.5_ exposure	8.25	3.88	2.12	**0.036**
RUCA	2.14	1.03	2.08	**0.040**
AGI	0.02	0.03	0.59	0.554

Change in total rhinologic subdomain score				
Intercept	−26.47	12.50	−2.11	**0.038**
Mean 12‐month PM_2.5_ exposure	1.95	1.50	1.30	0.198
RUCA	0.71	0.40	1.79	0.076
AGI	0.00	0.01	0.29	0.775

Change in total ENR subdomain score				
Intercept	−10.74	5.95	−1.80	0.07
Mean 12‐month PM_2.5_ exposure	0.82	0.71	1.15	0.25
RUCA	0.15	0.19	0.81	0.42
AGI	0.00	0.01	0.15	0.88

Change in total ear/facial subdomain score				
Intercept	−14.06	7.37	−1.91	0.060
Mean 12‐month PM_2.5_ exposure	1.12	0.88	1.27	0.206
RUCA	0.13	0.23	0.55	0.581
AGI	0.01	0.01	0.74	0.460

Change in total psychological subdomain score				
Intercept	−38.43	12.11	−3.17	**0.002**
Mean 12‐month PM_2.5_ exposure	3.69	1.45	2.54	**0.013**
RUCA	0.80	0.38	2.09	**0.040**
AGI	0.01	0.01	0.47	0.637

Change in total sleep subdomain score				
Intercept	−28.16	10.12	−2.78	**0.007**
Mean 12‐month PM_2.5_ exposure	2.57	1.21	2.12	**0.037**
RUCA	0.70	0.32	2.17	**0.033**
AGI	0.01	0.01	0.67	0.504

*Note*: Significant values are bolded.

Abbreviations: AGI, adjusted gross income; ENR, extra‐nasal rhinologic; PM, particulate matter; RUCA, rural–urban commuting area code; SE, standard error.

**TABLE 2:** Multivariate linear regression models of SNOT‐22 outcomes in chronic rhinosinusitis without nasal polyps.

Characteristic	*β*	SE	*t*‐test statistic	*p* value
Change in total SNOT‐22 score				
Intercept	−13.15	30.19	−0.44	0.664
Mean 12‐month PM_2.5_ exposure	−0.55	3.66	−0.15	0.881
RUCA	−0.24	1.48	−0.16	0.872
AGI	0.00	0.04	0.10	0.918

Change in total rhinologic subdomain score				
Intercept	−4.53	9.77	−0.46	0.644
Mean 12‐month PM_2.5_ exposure	−0.23	1.17	−0.20	0.846
RUCA	0.30	0.48	0.62	0.535
AGI	0.01	0.01	0.50	0.621

Change in total ENR subdomain score				
Intercept	2.02	5.50	0.37	0.714
Mean 12‐month PM_2.5_ exposure	−0.74	0.67	−1.11	0.271
RUCA	−0.03	0.27	−0.10	0.917
AGI	0.01	0.01	0.98	0.332

Change in total ear/facial subdomain score				
Intercept	−6.07	6.98	−0.87	0.387
Mean 12‐month PM_2.5_ exposure	0.28	0.85	0.33	0.740
RUCA	−0.03	0.34	−0.09	0.926
AGI	0.00	0.01	−0.01	0.996

Change in total psychological subdomain score				
Intercept	−7.99	10.58	−0.76	0.452
Mean 12‐month PM_2.5_ exposure	0.51	1.28	0.40	0.692
RUCA	−0.16	0.52	−0.30	0.764
AGI	−0.01	0.01	−0.75	0.456

Change in total sleep subdomain score				
Intercept	−4.03	9.60	−0.42	0.676
Mean 12‐month PM_2.5_ exposure	0.09	1.16	0.08	0.936
RUCA	−0.33	0.47	−0.71	0.483
AGI	−0.01	0.01	−0.58	0.561

*Note*: Significant values are bolded.

Abbreviations: AGI, adjusted gross income; ENR, extra‐nasal rhinologic; PM, particulate matter; RUCA, rural–urban commuting area code; SE, standard error.

## 4 Discussion

The results of this study suggest that chronic PM_2.5_ exposure is a potential risk factor for reduced QoL improvement after ESS in CRSwNP patients. The results of our multivariate regression identified that for each 1 µg/m^3^ increase in 12‐month PM_2.5_ exposure, there was a decrease in the magnitude of overall postoperative SNOT‐22 improvement by approximately 8 points, close to the published MCID of 8.9 points [9]. Within the psychological and sleep subdomains, an increase of 1 µg/m^3^ in 12‐month PM_2.5_ exposure was associated with a decrease in the magnitude of overall change by approximately 3 and 2 points, approaching the MCID values of these subdomains (3.2 and 2.9 points, respectively). These results indicate that across the total SNOT‐22 score and its subdomains, a 1–1.5 µg/m^3^ increase in exposure may impact postsurgical outcomes by a clinically meaningful amount [8].

It is notable that the two subdomains of the SNOT‐22 that appeared most impacted by estimated PM_2.5_ exposure were the psychological and sleep domains, which are generally considered to be secondarily impacted by CRS rather than primary hallmarks of the clinical syndrome. Prior studies have shown that patients with higher baseline scores among the psychological and sleep domains are more likely to pursue surgical management, though experience the smallest improvement in these symptoms postoperatively [10]. Based on our study, chronic PM_2.5_ exposure may further attenuate these improvements and be a driver of refractory symptomatology, highlighting the need for preoperative expectation counseling and the need for multifaceted management of these symptoms.

There are several important limitations to this study. First, our population consists of predominantly white patients located within a small geographic area, limiting its generalizability. In addition, though home addresses were confirmed at each appointment, our estimations may not accurately reflect individual exposures due to confounders such as occupational exposures or multiple addresses throughout the preoperative period. Smoking status was also not included due to low reliability in its collection. Lastly, while SNOT‐22 score was used as a measure of long‐term postoperative outcomes, this is often a noisy metric prone to within‐subject variation based on compliance with medications, acute exacerbations, and seasonality that were not accounted for.

Despite these limitations, the current study presents further evidence of the importance of air pollutant exposures and its association with poorer postoperative outcomes, meriting further investigation with larger multi‐institutional studies across a wider geographic range, potentially using wearable sensors to allow individualized exposure calculations.

## Conflicts of Interest

Katherine N. Cahill served on scientific advisory boards for AstraZeneca, Sanofi, Genentech, Regeneron, Novartis, served as a consultant for Eli Lilly, reports royalties from UpToDate, and reports research support from Novo Nordisk. Rakesh Chandra is a consultant to Sanofi, Regeneron, Optinose, Lyra Therapeutics. All other authors declare no conflicts of interest.

## Supporting information




**Supporting File 1**: alr70051‐sup‐0001‐TableS1.docx
