# Supplementary material for: The Impact of Chronic Particulate Matter Exposure on Quality‐of‐Life Outcomes After Endoscopic Sinus Surgery
Source: Int Forum Allergy Rhinol. 2025 Oct 21;15(12):1425–8. doi: 10.1002/alr.70051 (PMC12676652; doi:10.1002/alr.70051)
Supplement: Supplementary file 1 — Supporting File 1: alr70051‐sup‐0001‐TableS1.docx [file ALR-15-1425-s001.docx]

**Supplementary Table 1: Patient Demographics**

| **Characteristics** | **N (%)** | **Mean (SD)** | **Range** |
| --- | --- | --- | --- |
| Total Patients | 226 (100%) | - | - |
| CRSsNP | 113 (50%) | - | - |
| CRSwNP | 113 (50%) | - | - |
| Age | - | 50.5 (15.5) | (18-32) |
| Sex | - | - | - |
| Female | 115 (50.9%) | - | - |
| Male | 111 (49.1%) | - | - |
| Race |  |  |  |
| White | 190 (84.1%) | - | - |
| Black | 23 (10.2%) | - | - |
| Hispanic | 4 (1.8%) | - | - |
| Asian | 3 (1.3%) | - | - |
| Other | 6 (2.6%) | - | - |
| RUCA |  |  |  |
| 1 | 149 (65.9%) | - | - |
| 2 | 22 (9.7%) | - | - |
| 3 | 0 (0%) | - | - |
| 4+ | 38 (16.8%) | - | - |
| NA | 17 (7.5%) | - | - |
| Asthma |  |  |  |
| No | 126 (55.8%) | - | - |
| Yes | 100 (44.2) | - | - |
| Allergic Rhinitis |  |  |  |
| No | 100 (44.2%) | - | - |
| Yes | 126 (55.8%) | - | - |
| 12-Month PM 2.5 Exposure (µg/m3) |  | 8.2 (0.6) | (6.5-13.0) |
| Baseline SNOT-22 Score |  | 43.7 (19.9) | (1.0 - 95.0) |
| Preoperative Lund-Mackay CT Score |  | 15 (5.1) | (4.0-24.0) |

Abbreviations: SD = Standard Deviation, CRSsNP = Chronic Rhinosinusitis without Nasal Polyps, CRSwNP = Chronic Rhinosinusitis with Nasal Polyps, RUCA = Rural-Urban Commuting Area Code, PM = Particulate Matter
